# Chemokine-Driven Intercellular Crosstalk in the Osteosarcoma Microenvironment After Neoadjuvant Chemotherapy: A Single-Cell RNA Sequencing Study

**DOI:** 10.3390/biomedicines14091966

**Published:** 2026-08-31

**Authors:** Bangmin Wang, Jingyu Hou, Qilong Su, Jun Li, Weitao Yao

**Affiliations:** The Affiliated Cancer Hospital of Zhengzhou University & Henan Cancer Hospital, Zhengzhou 450008, China

**Keywords:** osteosarcoma, single-cell RNA sequencing, tumor microenvironment, neoadjuvant chemotherapy, CXCL2

## Abstract

**Background/Objectives:** Osteosarcoma (OS) is an aggressive bone malignancy with a complex tumor microenvironment (TME) that influences therapeutic outcomes and resistance. How neoadjuvant chemotherapy (NACT) reshapes the OS TME at single-cell resolution remains largely undefined. This study aimed to characterize cellular heterogeneity in the OS TME after NACT and identify chemokine-mediated intercellular crosstalk driving chemoresistance. **Methods:** Single-cell RNA sequencing was performed on surgical specimens from 12 OS patients (6 treatment-naive and 6 post-NACT). After quality control, 77,616 cells (36,214 from naive patients, 41,402 from post-NACT samples) were analyzed through the Seurat pipeline. Unsupervised clustering, differential expression analysis, and cell–cell communication network construction were performed, and candidate signaling axes were validated using transwell assays and Western blotting. **Results:** Cells were classified into 10 major cell types. Osteoblasts, identified as malignant cells, were partitioned into 11 subpopulations with marked transcriptional heterogeneity and differential PI3K/Akt pathway activity. Post-NACT, stromal and vascular components underwent molecular and functional remodeling, shaping an immune-activated microenvironment. Mononuclear phagocytes resolved into three discrete clusters—monocytes, macrophages, and dendritic cells—with differentiation gradients. Endothelial cells maintained robust *CXCL2* expression throughout the therapeutic course. Functional validation via transwell assays and Western blotting confirmed that endothelial-derived CXCL2 promoted macrophage chemotaxis via CXCR2, with corresponding CXCR2 upregulation in macrophages. **Conclusions:** Collectively, these findings suggest the complex cellular and transcriptional heterogeneity of the OS microenvironment and its chemokine-driven molecular remodeling after NACT, indicating that TME dynamics may be a determinant of therapeutic response and chemoresistance.

## 1. Introduction

Osteosarcoma (OS) represents the predominant primary malignant bone neoplasm within orthopedic practice [1]. Exhibiting a bimodal distribution, OS incidence peaks first during the adolescent growth spurt and subsequently in the elderly population. Arising from primitive mesenchymal osteoblasts, this malignancy frequently manifests in long bones, particularly the proximal tibia and distal femur [2], and is distinguished by aggressive behavior, high recurrence rates, and a propensity for distant metastasis [3]. The current standard of care for newly diagnosed OS encompasses neoadjuvant chemotherapy (NACT), surgical resection, and adjuvant chemotherapy [4,5]. Although NACT was introduced in the 1970s [6], contemporary protocols increasingly advocate for comprehensive regimens integrating NACT with preoperative radiotherapy [7,8]. While these advances have elevated the 5-year overall survival rate to approximately 65–70% [9], progress has plateaued in recent decades, likely due to the emergence of chemoresistance [10,11]. Standard NACT protocols typically employ high-dose methotrexate (MTX), doxorubicin (DOX), and cisplatin (DDP), administered sequentially or concurrently to ensure adequate dose intensity [12,13]. Although limb salvage and survival outcomes initially improved with optimized chemotherapy, the stagnation in long-term survival underscores the critical need to elucidate the molecular underpinnings of drug resistance, which remains mechanistically obscure and variable across patients. Consequently, identifying novel therapeutic targets to overcome resistance is imperative.

Emerging evidence suggests that the osteoblastic phenotype significantly shapes the tumor microenvironment (TME) by modulating immune cell infiltration and driving angiogenesis, processes pivotal to tumor progression and metastatic spread [14,15]. Moreover, the specific cellular architecture of the TME in osteoblastic OS has been correlated with chemotherapeutic efficacy, emphasizing the necessity of deciphering these complex interactions to engineer precise targeted therapies [16,17]. Given the distinct biological features of osteoblastic OS, rigorous investigation into its cellular dynamics and treatment responses is essential for enhancing patient prognoses and devising more potent therapeutic strategies. In this context, single-cell RNA sequencing (scRNA-seq) has emerged as a transformative tool for deep molecular profiling, capable of resolving genomic and transcriptomic alterations at the resolution of individual cells during tumorigenesis [18,19]. Recent technological strides in scRNA-seq offer unprecedented opportunities to map comprehensive cellular and molecular differentiation trajectories within tumor tissues [20], thereby serving as a robust platform for evaluating therapeutic efficacy and prognostic markers. To address the existing knowledge gaps, this study leverages scRNA-seq to dissect cellular heterogeneity and microenvironmental remodeling in OS, comparing primary tumors with those exposed to NACT. By examining individual cell transcriptomes, we aim to illuminate the intricate cellular crosstalk and variations within the TME, providing a refined reference for optimizing OS treatment paradigms.

## 2. Methods

### 2.1. Sample Selection Criteria and Collection

We collected samples from 19 patients with OS recruited at the Henan Provincial Cancer Hospital (Henan, China) under appropriate ethics approval (2021-KY-0172-002); Sample A4 did not include tumor tissue, so no follow-up experiments were performed. All 19 patients were diagnosed with osteoblastic OS according to the NCCN Clinical Practice Guidelines in Oncology (https://www.nccn.org/). Samples for scRNA-seq were derived from the primary tumor sites of 18 patients, six of whom had no prior oncological treatment, while 12 had received two cycles of NACT comprising a combination of DOX, DDP, and MTX. Informed consent was obtained prior to sample collection. Detailed information regarding patient clinical characteristics is provided in Table S1.

### 2.2. Tissue Dissociation and Single-Cell Suspension Preparation

Fresh tissues were stored in sCelLiveTM Tissue Preservation Solution (Singleron Biotechnologies, Nanjing, China) on ice immediately after collection via needle biopsy or surgery. The specimens were washed with Hanks’ balanced salt solution three times, minced into small pieces, and then digested with 3 mL sCelLiveTM Tissue Dissociation Solution (Singleron Biotechnologies, Nanjing, China) using a Singleron PythoN™ Tissue Dissociation System (SGR-TDAp10, Singleron Biotechnologies, Suzhou, China) at 37 °C for 15 min. The cell suspension was collected and filtered through a 40-µm sterile strainer. Afterwards, GEXSCOPE^®^ red blood cell lysis buffer (RCLB, Singleron Biotechnologies, Nanjing, China) was added, and the mixture [cell: RCLB = 1:2 (volume ratio)] was incubated at room temperature for 5–8 min to remove red blood cells. The mixture was then centrifuged at 300× *g* at 4 °C for 5 min to remove the supernatant and suspended softly in PBS. Finally, the samples were stained with Trypan Blue, and cell viability was evaluated microscopically.

### 2.3. ScRNA-Seq Library Preparation

Single-cell suspensions (2 × 10^5^ cells/mL) in PBS (HyClone) were loaded onto a microwell chip using the Singleron Matrix^®^ Single-Cell Processing System. Barcoding beads were subsequently collected from the microwell chip, followed by reverse transcription of the captured mRNA, cDNA synthesis, and PCR amplification. The amplified cDNA was fragmented and ligated using sequencing adapters. ScRNA-seq libraries were constructed according to the protocol of the GEXSCOPE^®^ Single-Cell RNA Library Kit (Singleron) [21]. Individual libraries were diluted to 4 nM, pooled, and sequenced on an Illumina NovaSeq 6000 (Illumina, San Diego, CA, USA) with 150 bp paired-end reads.

### 2.4. Generation of Single-Cell Gene Expression Matrices

Raw scRNA-seq reads were processed to generate gene expression matrices using the CeleScope (https://github.com/singleron-RD/CeleScope, accessed on 21 October 2022) v1.9.0 pipeline. Briefly, raw reads were first processed with CeleScope to remove low-quality reads using Cutadapt v1.17 [22] to trim the poly(A) tail and adapter sequences. Cell barcodes and unique molecular identifiers (UMIs) were extracted. We used STAR v2.6.1a [23] to map the reads to the reference genome GRCh38 (ensemble version 92 annotation). The UMI and gene counts of each cell were acquired using featureCounts v2.0.1 [24] software and used to generate expression matrix files for subsequent analysis.

### 2.5. Quality Control, Dimension Reduction, and Clustering

Cells were filtered by gene counts below 200, top 2% gene counts, and top 2% UMI counts. Cells with more than 20% mitochondrial content were excluded. After filtering, 77,616 cells were retained for downstream analyses with an average of 2495.825 genes and 8836.719 UMIs per cell. Functions from Seurat v3.1.2 [25] were used for dimension reduction and clustering. We then used the “NormalizeData” and “ScaleData” functions to normalize and scale all gene expressions and selected the top 2000 variable genes with the “FindVariableFeatures” function for principal component analysis. Using the top 20 principal components, the cells were separated into multiple clusters using FindCluster. The batch effect between the samples was removed using Harmony [26]. Finally, the uniform manifold approximation and projection (UMAP) algorithm was applied to visualize the cells in two-dimensional space.

### 2.6. Differentially Expressed Gene (DEG) Analysis

To identify DEGs, we used the Seurat “FindMarkers” function based on the Wilcox likelihood-ratio test with default parameters and selected genes expressed in >10% of the cells in a cluster with an average log (fold change) value >0.25. For cell type annotation of each cluster, we combined the expression of canonical markers in the DEGs with knowledge from the existing literature and displayed the expression of markers of each cell type with heatmaps/dot plots/violin plots generated using the Seurat “DoHeatmap”/“DotPlot”/“Vlnplot” functions, respectively. Doublets were identified as expression markers for different cell types and removed manually.

### 2.7. Cell Type Annotation

The cell type identity of each cluster was determined by the expression of canonical markers found in the DEGs using the SynEcoSys database. Violin plots displaying the expression of markers used to identify each cell type were generated using Seurat v3.1.2 “DoHeatmap”/“DotPlot”/“Vlnplot.”

### 2.8. Pathway Enrichment Analysis

To investigate potential DEG functions, Gene Ontology (GO) and Kyoto Encyclopedia of Genes and Genomes (KEGG) analyses were performed using the “clusterProfiler” package in R version 3.16.1 [27]. Pathways with adjusted *p* < 0.05 were considered significantly enriched. GO gene sets, including molecular function, biological process, and cellular component categories, were used as references. For gene set variation analysis (GSVA) pathway enrichment, the average gene expression of each cell type was used as input data using the “GSVA” package [28].

### 2.9. ScRNA-Seq-Based Copy Number Variation (CNV) Detection

The “InferCNV” package was used to detect CNVs in malignant OS cells [29]. Endothelial cells (ECs), erythrocytes, fibroblasts, mast cells, mononuclear phagocytes (MPs), mesenchymal stem cells (MSCs), mural cells, and T cells were used as baselines to estimate the CNVs of malignant cells. Genes expressed in more than 20 cells were sorted based on their chromosomal loci. The relative expression values were centered at 1, using 1.5 standard deviation from the residual-normalized expression values as the ceiling. A slide window size of 101 genes was used to smooth the relative expression on each chromosome to remove the effect of gene-specific expression.

### 2.10. Pseudotime Trajectory Analysis

Trajectory analysis of OS cells was performed using the Monocle 2 pipeline (version 2.18.0; http://github.com/cole-trapnell-lab/monocle3, accessed on 22 February 2023) in R software [30] with the following major parameters: mean expression >0.1 and num_cells_expressed ≥10. The “BEAM” function was used to examine DEGs between branches, and the “graph_test” function was used to identify co-expressed gene modules.

### 2.11. Cell–Cell Interaction Analysis

Cell–cell interaction was analyzed using CellPhoneDB v2.1.0 [31] based on known receptor–ligand interactions between two cell types/subtypes. The cluster labels of all cells were randomly permuted 1000 times to calculate the null distribution of the average ligand–receptor expression levels of the interacting clusters. Individual ligand or receptor expression was subjected to a threshold with a cut-off value based on the average log-gene expression distribution for all genes across all cell types. Significant cell–cell interactions were defined as *p* < 0.05 and average log expression >0.1, which were visualized using the “circlize” package in R version 0.4.10.

### 2.12. Transcription Factor (TF) Regulatory Network Analysis

A TF network was constructed in Pyscenic version 0.11.0 [32] using the scRNA expression matrix and TFs from AnimalTFDB. GRNBoost2 was used to predict a regulatory network based on the co-expression of regulators and targets. CisTarget was then used to exclude indirect targets and to search for TF-binding motifs. Subsequently, AUCell was used to quantify regulon activity in each cell. Top TF regulons with high regulon specificity score were visualized using “pheatmap” in R.

### 2.13. PMA-Induced Differentiation of THP-1 Monocytes into Macrophages

The human acute monocytic leukemia cell line (THP-1) was purchased through BNCC (Beijing, China) and cultured in RPMI-1640 with 10% FBS supplemented with 0.05 mM β-Mercaptoethanol. For macrophage differentiation, THP-1 cells were seeded at a density of 1 × 10^6^ cells/mL and incubated with 100 ng/mL phorbol 12-myristate 13-acetate (PMA; Cat No. CM00437, Proteintech, Wuhan, China) for 48 h at 37 °C in a humidified atmosphere of 5% CO_2_. Differentiation was confirmed by cell adherence and morphological changes, and further validated by flow cytometric analysis of macrophage markers (e.g., CD11b and CD68). After differentiation, cells were washed twice with PBS and used in subsequent experiments.

### 2.14. Plasmid Construction and Stable Overexpression in HUVECs

X-C motif chemokine ligand 2 (*CXCL2*)-overexpressing plasmid was constructed by Nanjing Kerui Biotechnology Co., Ltd. (Nanjing, China). The target gene encoding *CXCL2* was obtained by gene synthesis. The fragment was cloned into the pLV11ltr-PGK-Puro(2A)mCherry-CMV-c3xFlag vector using XhoI and XbaI restriction sites. Human umbilical vein endothelial cells (HUVECs) were purchased from BNCC (Beijing, China) and cultivated after manufacturer’s instructions. HUVECs were transfected with the plasmid and selected with puromycin (2 μg/mL) for 48 h to obtain stable overexpression.

### 2.15. Reagent Preparation and Chemotaxis Assay

Recombinant human GRO-β/CXCL2 protein (Cat. No. HY-P7190, MedChemExpress, Shanghai, China) was reconstituted in sterile deionized water to a stock concentration of 100 μg/mL and stored at −20 °C. For the transwell migration assay, THP-1-derived macrophages were starved in serum-free medium for 4–6 h and then seeded into the upper chamber (1 × 10^5^ cells per insert, 5-μm pore size) in 100 μL of serum-free medium. To determine the optimal chemotactic concentration, a gradient of recombinant CXCL2 (5–100 ng/mL) was tested in the lower chamber (filled with 600 μL of serum-free medium). Based on the migration response, 25 ng/mL CXCL2 was selected for subsequent experiments. After incubation at 37 °C for 24 h, non-migrated cells on the upper membrane surface were gently removed with a cotton swab. Migrated cells were fixed with 4% paraformaldehyde, stained with 0.1% crystal violet, and counted under a light microscope at 100× magnification. For endothelial cell-derived CXCL2 chemotaxis assays, *CXCL2*-overexpressing HUVECs were plated in the lower chamber and induced to express *CXCL2* for 24 h before the transwell assay.

### 2.16. Western Blot Analysis for CXCR2 Expression

The expression of C-X-C Motif Chemokine Receptor 2 (CXCR2) in macrophages under different treatment conditions (CXCL2 stimulation and endothelial cell overexpression) was assessed by Western blotting. Total protein lysates were separated by SDS-PAGE and transferred onto PVDF membranes. Membranes were blocked with 5% non-fat milk in TBST for 1 h at room temperature, then incubated overnight at 4 °C with a rabbit polyclonal anti-CXCR2 antibody (Cat No. 20634-1-AP, Proteintech, Wuhan, China; diluted 1:1000 in blocking buffer). After washing, membranes were incubated with HRP-conjugated goat anti-rabbit secondary antibody (1:5000) for 1 h at room temperature. Protein bands were visualized using enhanced chemiluminescence (ECL) and quantified by densitometry. Equal loading was verified by reprobing the membranes with a rabbit polyclonal anti-GAPDH antibody (Cat No. 10494-1-AP, Proteintech; diluted 1:5000).

### 2.17. Statistics and Repeatability

Cell distribution between two groups was compared using an unpaired two-tailed Wilcoxon rank-sum test. Gene expression or gene signatures between two cell groups were compared using a Wilcoxon rank-sum test. Cell distribution of paired before-NACT and after-NACT groups was compared using paired two-tailed Wilcoxon rank-sum tests. All statistical analyses and presentations were performed using R. Statistical tests used in figures are indicated in figure legends, and statistical significance was set at *p* < 0.05. The exact *n* values and what they represent are shown in the figures and figure legends.

## 3. Results

### 3.1. High-Resolution scRNA-Seq Revealed the Landscape of OS Lesions

**Figure 1:**
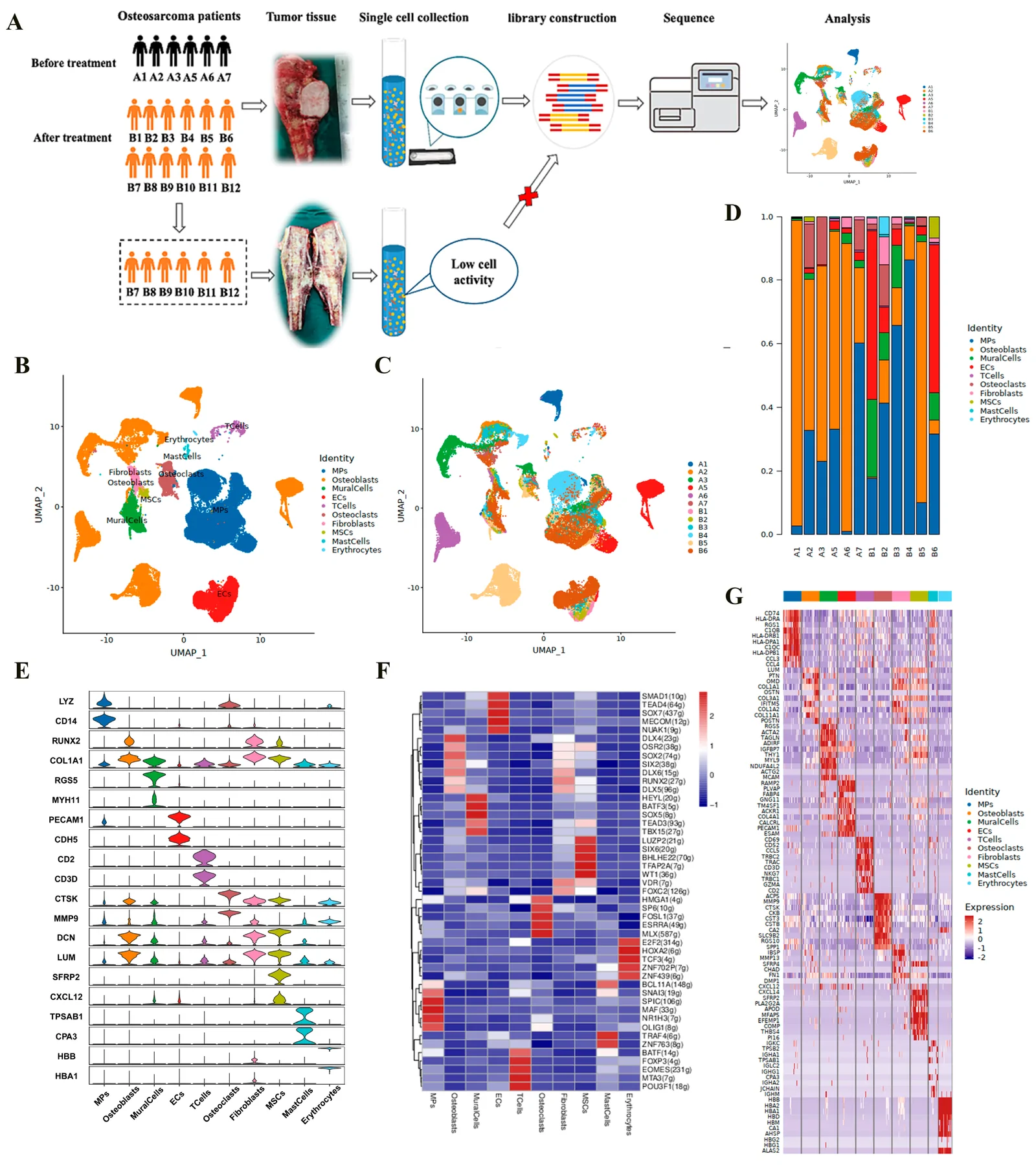
High-resolution scRNA-seq revealed the landscape of OS lesions. (**A**) Flow chart; (**B**) UMAP plot showing all identified cell types. (**C**) UMAP plot coloring by samples. (**D**) Bar chart of cell type proportions for 12 samples. (**E**) Violin diagram of 10 cell type marker genes. (**F**) Heatmap of SCENIC individual cells regulating gene expression through transcription factors. (**G**) Heatmap showing the expression of different cell type-specific marker genes.

Tumor tissues were obtained from six treatment-naive patients, including five puncture biopsy tumor samples and one surgically resected tumor sample, as well as resection specimens from 12 patients who had received NACT (Figure 1A, Table S1). The cell viability of six samples was low after dissociating the cells into cell suspension, so the experimental procedure was terminated. We performed scRNA-seq analysis of 12 OS samples. After initial quality control assessment and doublet removal, we obtained single-cell transcriptomes from 77,616 cells, including 36,214 cells from treatment-naïve patients and 41,402 cells from tumor samples surgically removed after NACT. The number of detected unique molecular indexes ranged from 4556 to 12,286 per cell, with the mean number of detected genes ranging from 1500 to 3089 (Figure S1A).

Unbiased cell clustering identified 10 main clusters in parallel based on UMAP analyses according to their gene profiles and canonical markers (Figure 1B). The cell type clusters, such as osteoblasts, MPs, and ECs, were significantly different between the samples (Figure 1C,D and Figure S1C). Similar to previous observations, stromal and immune cells from different patients or groups clustered together by cell types, whereas cancer cells showed higher heterogeneity and patient-specific expression signatures. Among these clusters, the most prominent differences were seen in: (1) osteoblastic OS cells highly expressing *RUNX2*, *COL1A1*, and *LUM*; (2) MPs, including macrophages, monocytes, and conventional dendritic cells, characterized by high *LYZ*, *C1QA*, *MRC1*, *CD14*, *VCAN*, *CD1C*, and *CLEC10A* expression; (3) ECs that specifically express the markers *PECAM1*, *CDH5*, *PLVAP*, and *ENG*; (4) osteoclast cells highly expressing *CTSK*, *MMP9*, and *ACP5*; (5) mural cells with high *RGS5*, *ACTA2*, *MCAM*, and *MYH11* expression; (6) fibroblasts specifically expressing *DCN*, *LUM*, and *COL1A1*; (7) MSCs expressing *SFRP2*, *CXCL12*, *THY1*, *IGFBP2*, and *MME*; (8) T cells highly expressing *CD2*, *CD3D*, *TRAC*, and *TRCB*; (9) erythrocytes expressing *HBB*, *HBA1*, *ALAS2*, and *SNCA*; (10) mast cells specifically expressing *TPSAB1*, *TPSB2*, and *CPA3*. The expression profiles of representative genes in the cell populations are shown in Figure 1E. The top 20 OS marker genes for each cell type were visualized as heat maps (Figure 1G). Single-cell regulatory network inference and clustering (SCENIC) analysis was performed to determine the TFs in the OS cells. *Runx2* was highly expressed in osteoblasts. Runx2, also known as Cbfa1, is a key TF that regulates the osteogenic differentiation of stem cells. Runx2 regulates the expression of bone *ALP*, a gene involved in early osteoblast differentiation, and promotes the differentiation of osteoblast progenitor cells into osteoblast precursor cells. The genes of three TFs (*SPIC*, *MAF*, and *NR1H3*) were significantly activated in MPs, three TFs (*BATF*, *FOXP3*, and *Eomes*) were activated in T cells, and two TFs (*SMAD1* and *SOX7*) were significantly activated in ECs (Figure 1F and Figure S1B).

To evaluate whether malignant cells demonstrate heterogeneity driven by somatic genome alterations, we inferred CNVs from our scRNA-seq data using the “InferCNV” algorithm (see the “2.9” for details). Expression differences across chromosomal segments were used to predict CNV regions. We further confirmed that malignant cells had more copy number expansion signatures and deletions than the reference cells. Heatmaps illustrating the relative expression intensities across each chromosome in OS cells were prepared. Most cells showed relatively low CNV levels, except for osteoblasts, whose eight subclones experienced remarkable CNV events compared with the reference cells. Notably, subclone 3 presented significantly higher CNV levels (Figure S1D). This analysis revealed extensive CNVs in all osteoblasts, suggesting that all osteoblasts are malignant in OS lesions.

### 3.2. The OS Microenvironment Comprises Multiple Cell Subtypes, Which Are Differentially Modulated by the Response to NACT

**Figure 2:**
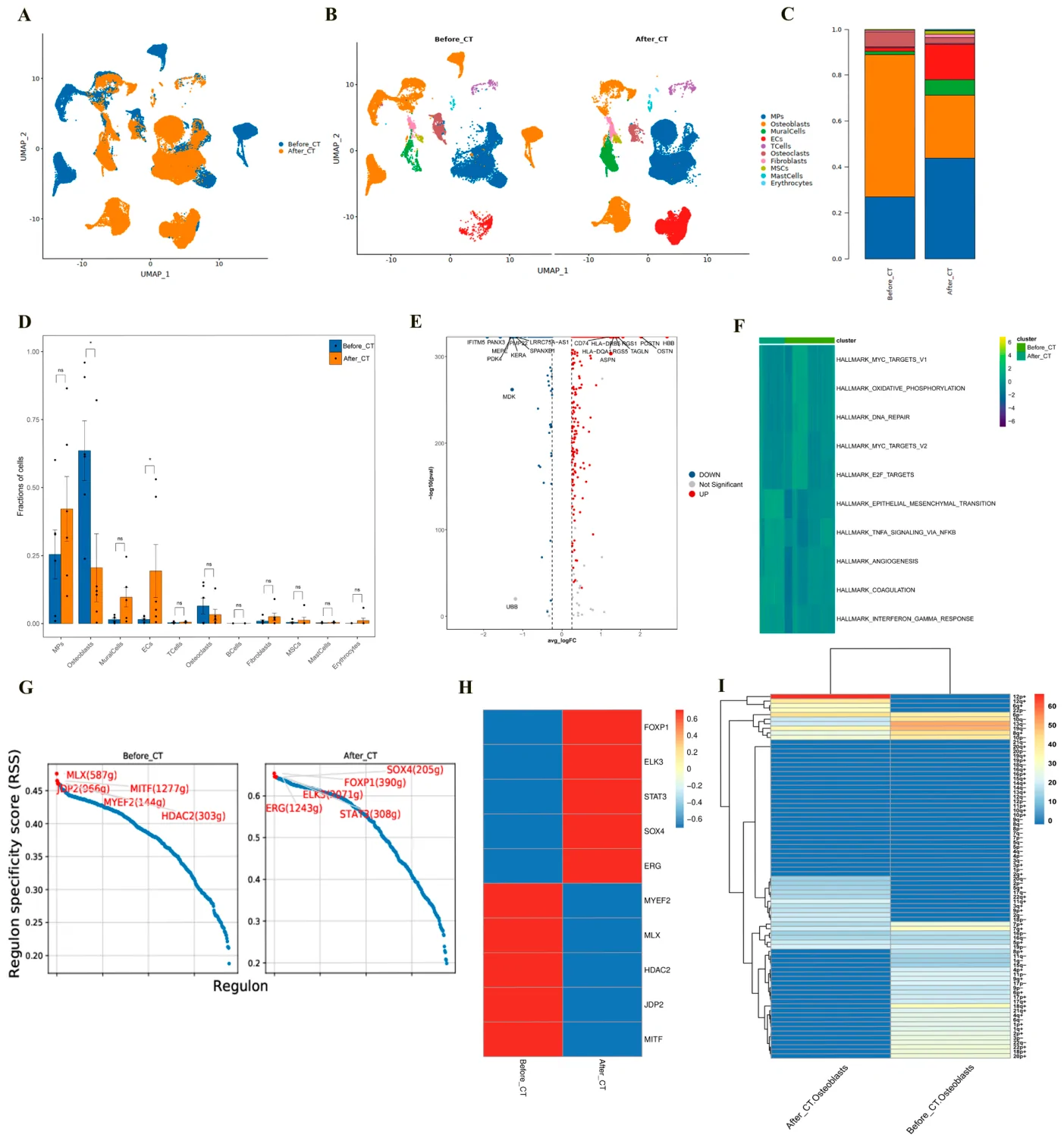
Differences in cellular and genetic levels before and after OS NACT. (**A**) UMAP plot coloring before and after NACT. (**B**) UMAP plot showing different cell types before and after NACT; (**C**) Bar graph of cell type ratios before and after NACT. (**D**) Differences in individual cell ratios before and after NACT, * *p* < 0.05. (**E**) Display of differential genes before and after NACT, showing only the top 10 up- and down-regulated genes. (**F**) Differences in HALLMARK pathway levels before and after NACT. (**G**) Before and after NACT specificity-related transcription factors, horizontal coordinates indicate rankings, vertical coordinates indicate RSS specificity scores and the top five Regulon rankings are indicated by red dots. (**H**) Heatmap of SCENIC before and after NACT for regulation of gene expression through transcription factors. (**I**) Osteoblasts before and after NACT at different chromosome amplification deletion states, vertical coordinates are the amplification deletion states expressed by the long and short arms of chromosomes (long arm (q), short arm (p)), numbers represent different chromosomes.

UMAP profiles and cell type contexts were stratified before or after NACT to allow transcriptional comparison of homologous populations in different clinical settings (Figure 2A). The total identified cell numbers and proportions from the different groups are provided (Figure 2B). When analyzing cell type abundance post-treatment, we observed significantly fewer osteoblasts and more ECs in the post-treatment samples than in pre-treatment samples (*p* < 0.05) (Figure 2C,D), whereas the other cell types showed no significant change.

Drug-induced changes may reveal important insights into chemotherapeutic molecular mechanisms. Therefore, we investigated the global gene expression profiles of cells before and after treatment. A volcano plot of DEGs between the two groups was prepared. The difference between the percentages of groups expressed in the two clusters was plotted against the log-fold change in the average expression (Figure 2E). *CD74*, *HLA-DRB5*, *RGS1*, *POSTN*, *HBB*, *HLA-DQA1*, *RGS5*, *TAGLN*, *OSTN*, and *ASPN* were upregulated after NACT, whereas *IFITM5*, *PANX3*, *PMP22*, *LRRC75A-AS1*, *MEPE*, *SPANXB1*, *KERA*, *RDK4*, and *MDK* were downregulated. To investigate how NACT affects cells, we compared pathway activation in cells before and after NACT using GSVA. Gene set enrichment analysis results of the top four hallmark genes revealed enriched DEGs associated with genes in “HALLMARK_EPITHELIAL_MESENCHYMAL_TRANSITION,” “HALLMARK_ TNFA_SIGNALING_VIA_NFKB,” “HALLMARK_ANGIOGENESIS,” “HALLMARK_COAGULATION,” and “HALLMARK_INTERFERON_GAMMA_RESPONSE.” These results indicated that DEGs were closely related to TNFA signaling via NFKB, epithelial–mesenchymal transition (EMT), and angiogenesis. INTERFERON_GAMMA_RESPONSE pathway upregulation after treatment suggests the importance of interferon-γ response genes in the bioactivity of immunoreactive responses induced by NACT (Figure 2F). GO/KEGG functional enrichment results are presented in Figure S1E,F. To further dissect the transcriptional heterogeneity of gene regulation, SCENIC analysis of DEGs was performed to assess differences in TF expression levels before and after NACT. Further analysis showed that *FOXP1*, *SOX4* and *ERG* were specifically expressed in the NACT group, whereas *MYEF2* and *MLX* were activated only in the pre-NACT group (Figure 2G,H).

Next, we used CNVs to probe the clonal structure of each tumor. The CNV content of individual cells was ascertained from scRNA-seq data using InferCNV. OS was characterized by chromosomal instability, where chromosomes or parts of chromosomes with high levels of somatic structural variations and CNVs were duplicated or deleted. Recurrent regions of amplification and DNA copy number gain as well as DNA deletion or loss-of-heterozygosity are shown in Figure 2I.

### 3.3. Transcriptional Heterogeneity of Malignant OS Cells Due to NACT

**Figure 3:**
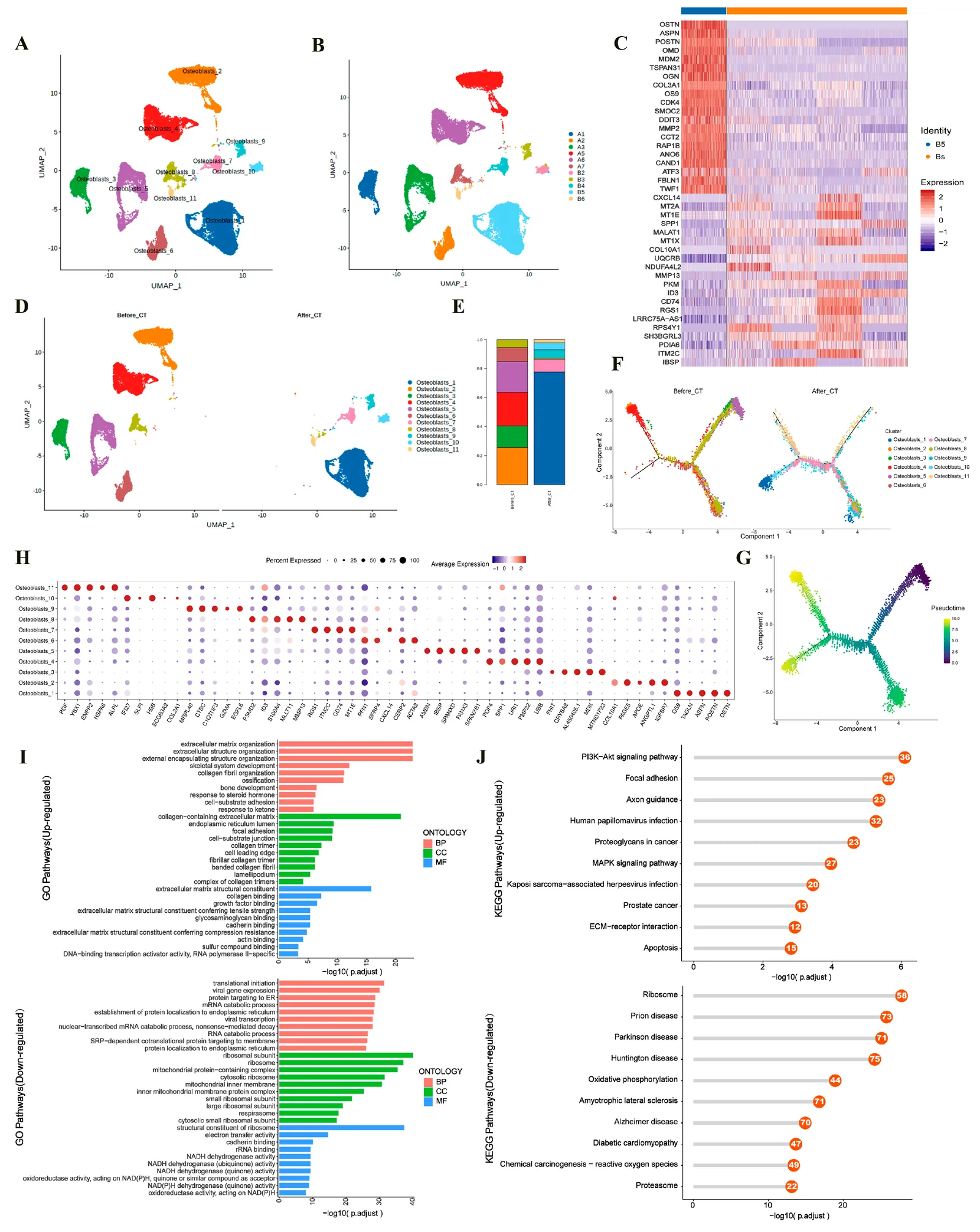
Resubdivision of osteoblasts into subpopulations. (**A**) UMAP plot showing osteoblast subpopulations. (**B**) UMAP plot showing osteoblast samples; (**C**) Display of differential genes in sample B5 and other samples after NACT, showing only the top 10 up and down-regulated genes. (**D**) UMAP plot showing different osteoblast types before and after NACT. (**E**) Bar graph of osteoblast type ratios before and after NACT. (**F**,**G**) Display the proposed chronological diagram of osteoblasts, the sequence of time indicates the sequence of pseudo-times of differentiation and distribution of different osteoblast types with pseudo-time before and after NACT. (**H**) Bubble plot showing the marker genes of osteoblast subtypes. (**I**) The up- and down-regulated GO functional pathways enriched by DEGs between sample B5 and other samples after NACT. The upper panel represents up-regulated pathways, and the lower panel represents down-regulated pathways. (**J**) The up- and down-regulated KEGG pathways enriched by DEGs between sample B5 and other samples after NACT. The upper panel represents up-regulated pathways, and the lower panel represents down-regulated pathways.

Osteoblastic OS is a major type of conventional OS. To better understand osteoblast transcriptional features, we re-clustered and characterized them into 11 different clusters (Figure 3A). Remarkably, the malignant sub-clusters were highly patient-specific, suggesting prominent molecular inter-tumor heterogeneity in the OS samples, whereas the proportion of each cell type varied greatly by sample (Figure 3B and Figure S2A). A UMAP plot presents the osteoblastic subpopulations in the two groups, colored to show cluster information. The clustering results of the UMAP osteoblast analysis revealed strong transcriptional heterogeneity before and after treatment. Osteoblasts_1, osteoblasts_7, osteoblasts_9, osteoblasts_10, and osteoblasts_11 corresponded to patients after NACT, and the other OS cell clusters were mainly from patients before NACT (Figure 3D,E). We analyzed the scRNA-seq data using Monocle 2, which reconstructs putative branching transcriptional trajectories to identify potential relationships across the calculated states (Figure 3F,G). We found two distinct evolutionary branches in the pre-treatment samples, but no significant evolutionary relationship was found in post-treatment cells. In the pre-treatment samples, osteoblasts_3 and osteoblasts_5 were the starting points, followed by osteoblasts_8, osteoblasts_2, and osteoblasts_5, with osteoblasts_1 occupying the terminal end of the pseudotime trajectory. Interestingly, osteoblasts_1 displayed high expression levels of the extracellular matrix proteins POSTN and OSTN. Osteoblasts_2 was characterized by high expression levels of mesenchymal markers, such as *COL10A1*, indicating EMT characteristics. Osteoblasts_8 was characterized by high expression levels of *MMP13* and *S100A4*, also known as fibroblast-specific protein 1. Osteoblasts_7, mainly including patients after NACT, exhibited high expression levels of the immune-associated genes *CD74* and *HLA-DRA* (Figure 3H). We observed a significant osteoblasts_1 subpopulation in B5 samples, so we extracted data from these and other NACT-treated samples for DEG analysis and functional enrichment. Moreover, the differences between the B5 samples and other NACT-treated samples were analyzed, and 1536 DEGs were obtained; the top 20 significantly upregulated and downregulated DEGs are presented as a heatmap (Figure 3C). The upregulated DEGs were *OSTN*, *ASPN*, *POSTN*, *OMD*, *MDM2*, *TSPAN31*, *OGN*, *COL3A1*, *OS9*, *CDK4*, and others, whereas the downregulated DEGs were *CXCL14*, *MT2A*, *MT1E*, *SPP1*, *MALAT1*, *MT1X*, *COL10A1*, *UQCRB*, *NDUFA4L2*, *MMP13*, *PKM*, and others (Figure 3C). GO enrichment analysis of these genes was conducted, and the 30 most significant GO items are plotted in Figure 3I. DEG enrichment was assessed using KEGG pathway analysis, and the upregulated genes were mainly enriched in pathways involving signal transduction, apoptosis, tumor necrosis factor, and tumor-associated proteins (Figure 3J). Notably, after chemotherapy, the upregulated genes were significantly enriched in the PI3K/Akt signaling pathway.

### 3.4. Single-Cell Profiling of Myeloid Cells in OS and Association with NACT Response

**Figure 4:**
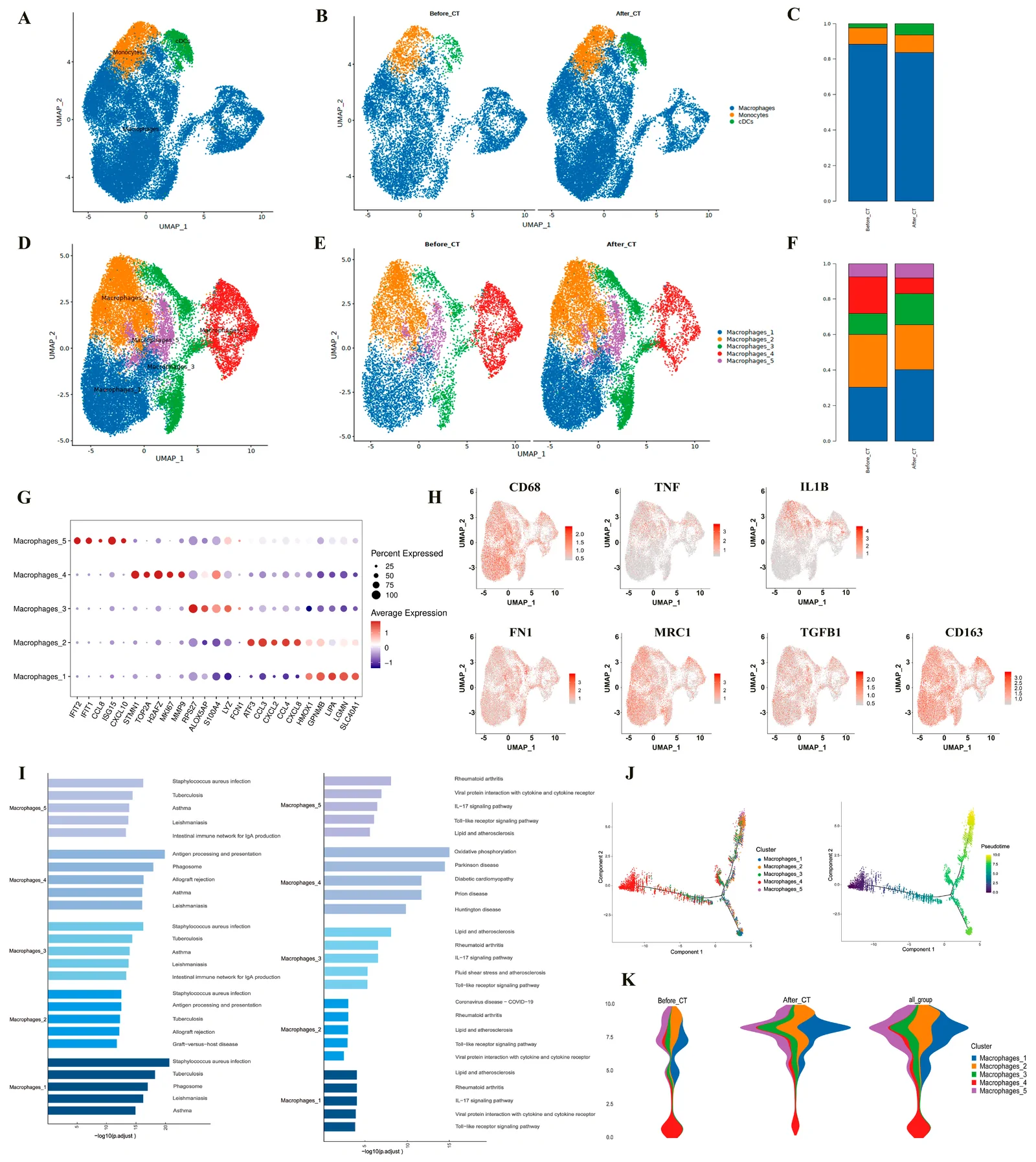
Mononuclear phagocyte subpopulation segmentation and proposed time series analysis. (**A**) UMAP plot showing mononuclear phagocyte subpopulations. (**B**) UMAP plot showing different mononuclear phagocyte types before and after NACT. (**C**) Bar graph of mononuclear phagocyte type ratios before and after NACT. (**D**) UMAP plot showing macrophage subpopulations. (**E**) UMAP plot showing different macrophage types before and after NACT. (**F**) Bar graph of macrophage type ratios before and after NACT. (**G**) Bubble map of macrophage type marker genes. (**H**) UMAP plot showing M1/M2 marker genes. (**I**) KEGG enrichment analysis of different macrophage types. (**J**) Display the proposed chronological diagram of macrophages, the sequence of time indicates the sequence of pseudo-times of differentiation and distribution of different macrophage types with pseudo-time. (**K**) The proportion of macrophages clusters at different time points before and after NACT, the ordinate from bottom to top is the pseudo-time sequence, the abscissa is the proportion of cell types at different time points, and different colors represent different cell types.

Tumor-infiltrating myeloid cells are highly heterogeneous and play important roles in immune evasion and response to immunotherapy. We investigated TME characteristics in OS. In this study, we focused on MPs and analyzed their different cell clusters in detail. Therefore, these clusters were selected for further investigation. Analysis of 27,836 MPs revealed three distinct subclusters: monocytes, macrophages, and dendritic cells. For OS lesions, monocytes and macrophages accounted for 70–90% of all MPs, while a minority were identified as dendritic cells (Figure 4A–C).

Further analysis of the 23,746 macrophages revealed five clusters (macrophages_1–5) with different marker gene expression before and after NACT (Figure 4D–G). Although all five subpopulations were enriched in both groups, cell abundance in the NACT group increased significantly, except for macrophages_4. Macrophages are classified into two canonical subtypes: proinflammatory M1 and anti-inflammatory M2 [33,34,35]. However, we could not clearly distinguish M1 and M2 macrophages using known marker genes, such as *CD68*, *IL1B*, and *TNF* (M1), and *MRC1*, *TNFB1*, *CD163*, and *FN1* (M2), as they were all expressed in macrophages_1–5. Next, we analyzed the expression of these marker genes between the two groups before and after NACT, suggesting that the macrophage polarization state changed after NACT. Tumor-associated macrophage (TAM) markers, as well as *CD68* and *CD163*, which are both M1/M2 and TAM markers, were highly expressed in each macrophage subtype, indicating that all subtypes were TAMs (Figure 4H). We investigated DEGs and enrichment of the five macrophage subpopulations in the different groups. KEGG enrichment analysis revealed distinct metabolic pathways in these clusters, showing downregulated macrophages_1, macrophages_3, and macrophages_5 genes in the IL-17 signaling pathway (Figure 4I). IL-17 is a highly versatile pro-inflammatory cytokine crucial for a variety of processes, including host defense, tissue repair, inflammatory disease pathogenesis, and cancer progression.

We further explored dynamic cell transitions by inferring state trajectories using the Monocle software. This pseudotime analysis revealed that macrophages_4 were present at the beginning of the trajectory path, whereas macrophages_1 and macrophages_2 were present at two different terminal stages (Figure 4J,K).

### 3.5. Chemotherapy-Induced EC Subpopulation Dynamics and Their Intercellular Communication with Macrophages in the TME

**Figure 5:**
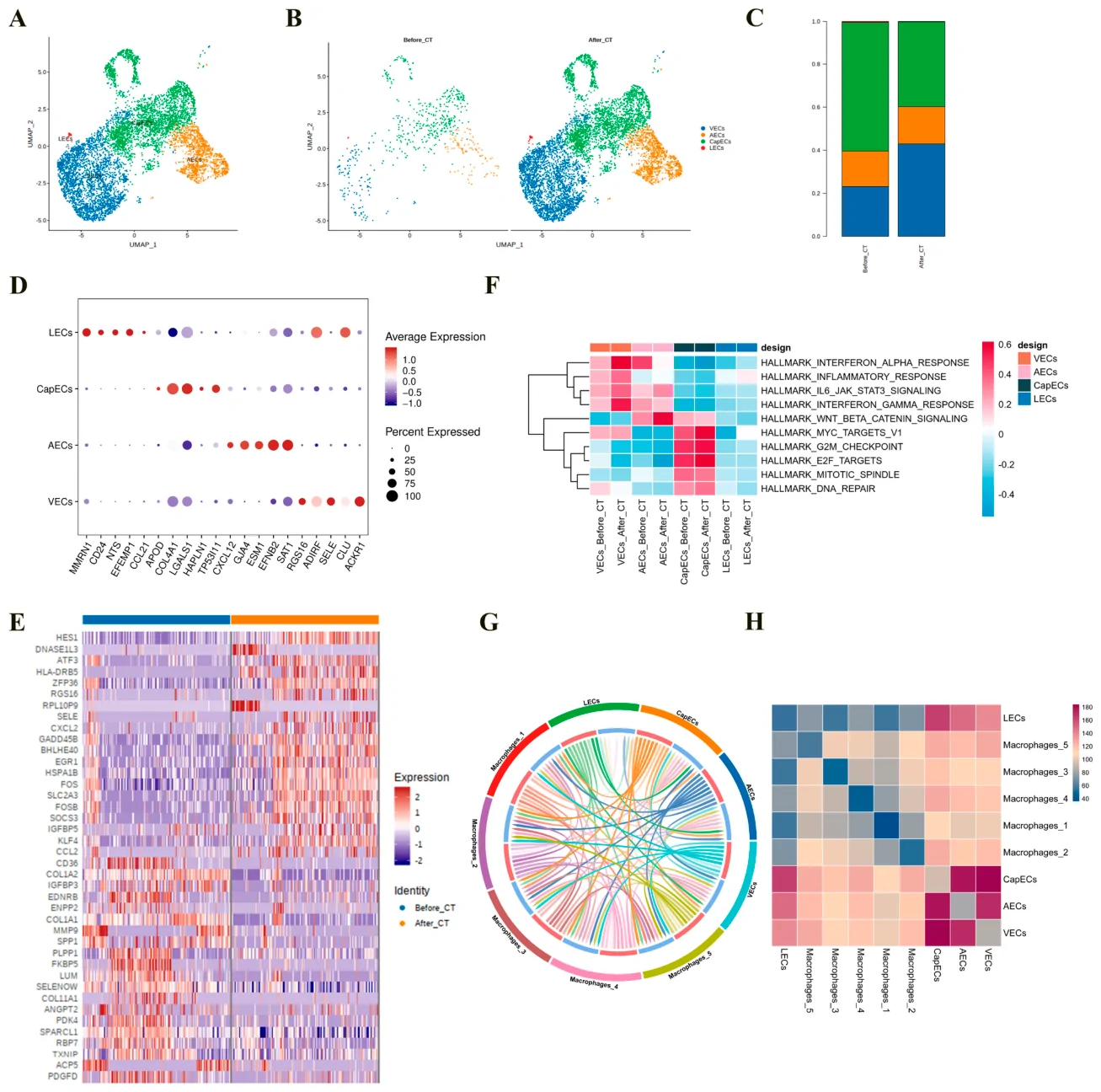
Gene expression patterns and intercellular communication among ECs and macrophage subpopulations. (**A**) UMAP plot showing EC subpopulations. (**B**) UMAP plot showing different EC types before and after NACT. (**C**) Bar graph of EC type ratios before and after NACT. (**D**) Bubble map of EC types’ marker genes. (**E**) Display of differential genes before and after NACT in ECs. (**F**) Differences in the levels of HALLMARK pathway in different EC types. (**G**) Circos plot showing intercellular interaction networks between EC subpopulations and macrophage subpopulations. (**H**) Heatmap of correlation between EC subpopulations and macrophage subpopulations.

A total of 6939 ECs were selected and re-clustered to characterize stromal cell dynamics in the TME. *PECAM1*, *CDH5*, *PLVAP*, and *ENG* expression levels were used to identify ECs. Four EC subpopulations were identified via unsupervised clustering of 6939 cells, including arteriole ECs, capillary endothelial cells (CapECs), venule endothelial cells (VECs), and lymphatic-type ECs based on classical EC markers (Figure 5A). As indicated earlier, the overall EC proportion was markedly increased within the TME. EC sub-clusters were predominantly identified in NACT samples. In the post-NACT group, the proportion of CapECs was low, whereas the number of VECs was elevated (Figure 5B,C). Details of the marker genes for each cell type are shown in Figure 5D. Next, we investigated gene expression levels in ECs (Figure 5E). Subsequently, we explored the HALLMARK pathway enrichment between the two groups using the GSVA method (Figure 5F). GSVA/hallmark pathway analysis revealed that significant upregulation of interferon vascular ECs are also important TME components during OS growth and metastasis. However, the interaction between vascular ECs and OS and its mechanism have not been fully elucidated. Therefore, it is very important to explore the key molecules in the EC-OS interaction and their roles and molecular mechanisms. Interferon-alpha, interferon-gamma, and inflammatory responses increased in the NACT group, indicating enhanced anti-tumor immunity of VECs. DEG analysis revealed a significant upregulation of chemokines after chemotherapy, suggesting potential remodeling of the TME. As chemokines are critical for endothelial–immune cell communication, we further focused on interactions between ECs and macrophages. We visualized the crosstalk among endothelial and macrophage subpopulations using Circos plots, where lines represent ligand–receptor interactions or communication relationships (Figure 5G). This analysis unveiled inter-subpopulation communication patterns, identifying which endothelial subsets (e.g., LECs, CapECs, AECs, VECs) engaged in strong interactions with particular macrophage groups. Additionally, a heatmap revealed the transcriptional correlations between endothelial subtypes and macrophage subtypes (Macrophages_1–5). Notably, LECs exhibited a negative correlation with nearly all macrophage subtypes, whereas CapECs, AECs, and VECs showed positive correlations with most macrophage subtypes (Figure 5H). These findings together provide insights into the intercellular regulation and molecular basis of endothelial–macrophage interactions.

### 3.6. Endothelial-Derived CXCL2 and Macrophage CXCR2 Drive Intercellular Interactions in the TME

**Figure 6:**
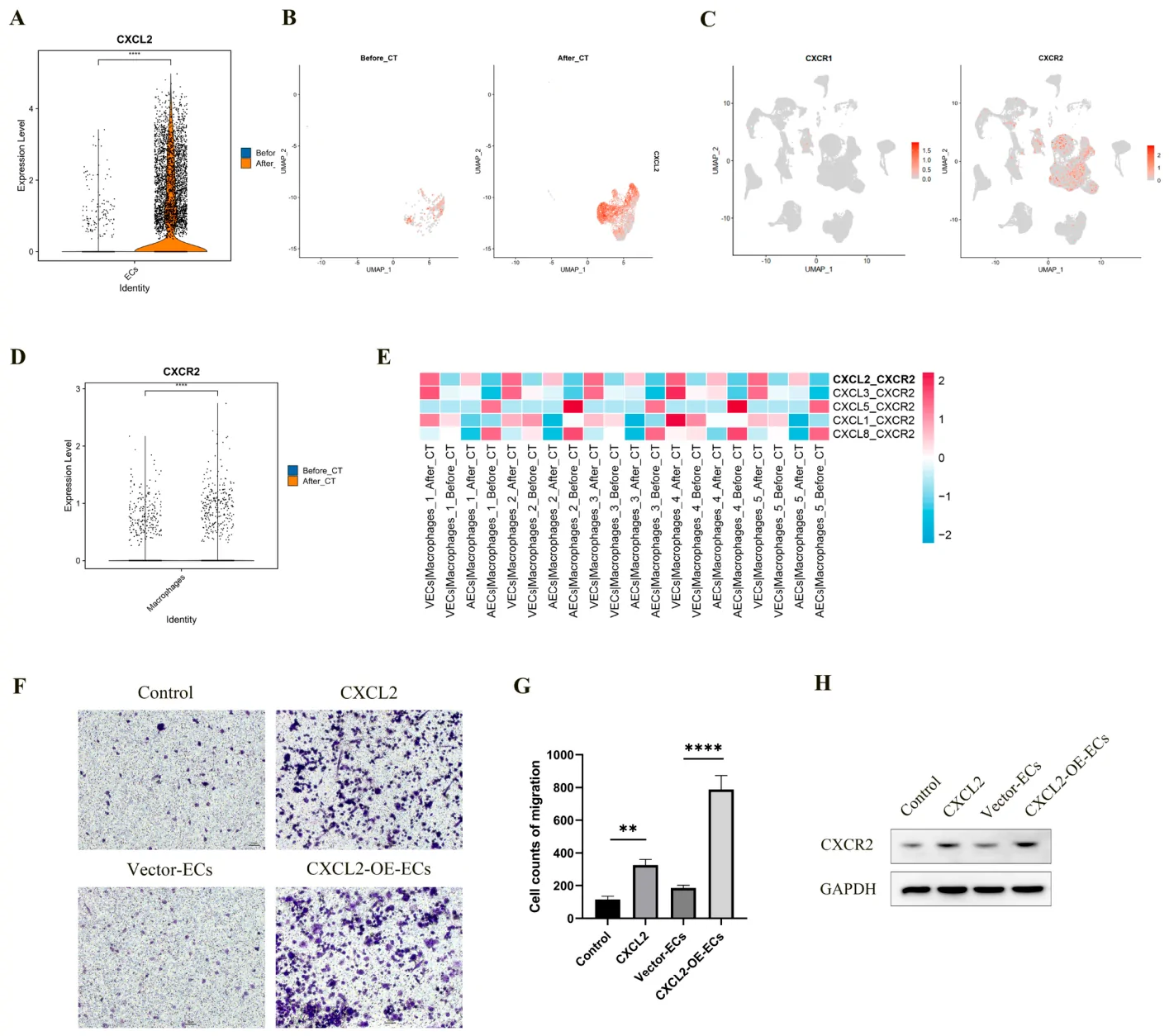
Cell-Type-Specific Expression, Distribution, and Functional Roles of CXCL2 and Its Receptor CXCR2. (**A**) Violin plot of *CXCL2* expression levels in ECs before and after NACT. (**B**) UMAP embeddings overlaid with *CXCL2* expression in ECs before and after NACT. (**C**) UMAP visualization of *CXCR1* and *CXCR2* expression distribution. (**D**) Violin plot showing *CXCR2* expression levels in macrophages before and after NACT. (**E**) Heatmap showing correlations between macrophage *CXCR2* receptor and EC ligand gene expression before and after NACT. (**F**) Transwell assay showing macrophage migration cell counts in different groups. (**G**) Bar plot showing quantitative analysis of migrated macrophages, ** (*p* < 0.01) **** (*p* < 0.0001). (**H**) Western blot validation of CXCR2 expression in different groups.

To further explore the molecular mediators of this crosstalk, we focused on chemokine signaling, which plays a critical role in intercellular communication within the TME. Notably, ECs exhibited significant expression of the chemokine *CXCL2*, which was markedly altered after NACT (Figure 6A,B). Among its receptors, *CXCR1* and *CXCR2* showed distinct expression patterns across cell types (Figure 6C), with CXCR2 being the predominant receptor on macrophages. *CXCR2* expression was significantly upregulated in macrophages following NACT (Figure 6D). Moreover, a heatmap analysis revealed strong correlations between macrophage *CXCR2* and multiple EC-derived ligand genes before and after treatment (Figure 6E), further supporting the importance of the CXCL2–CXCR2 axis. Functional validation using transwell assays showed that macrophage migration was significantly enhanced upon stimulation with recombinant CXCL2. Furthermore, endothelial cells overexpressing *CXCL2* promoted macrophage chemotaxis toward them, confirming the functional activity of endothelial-derived *CXCL2* (Figure 6F). Quantitative analysis confirmed statistically significant differences (** *p* < 0.01, **** *p* < 0.0001) (Figure 6G). Western blot analysis confirmed corresponding changes in CXCR2 protein levels (Figure 6H). Collectively, these results underscore the critical role of the CXCL2–CXCR2 signaling axis in mediating endothelial–macrophage communication and its modulation by chemotherapy.

### 3.7. NACT-Driven Fibroblast Subclusters and Osteoclast Phenotypic Transitions Remodel the TME

**Figure 7:**
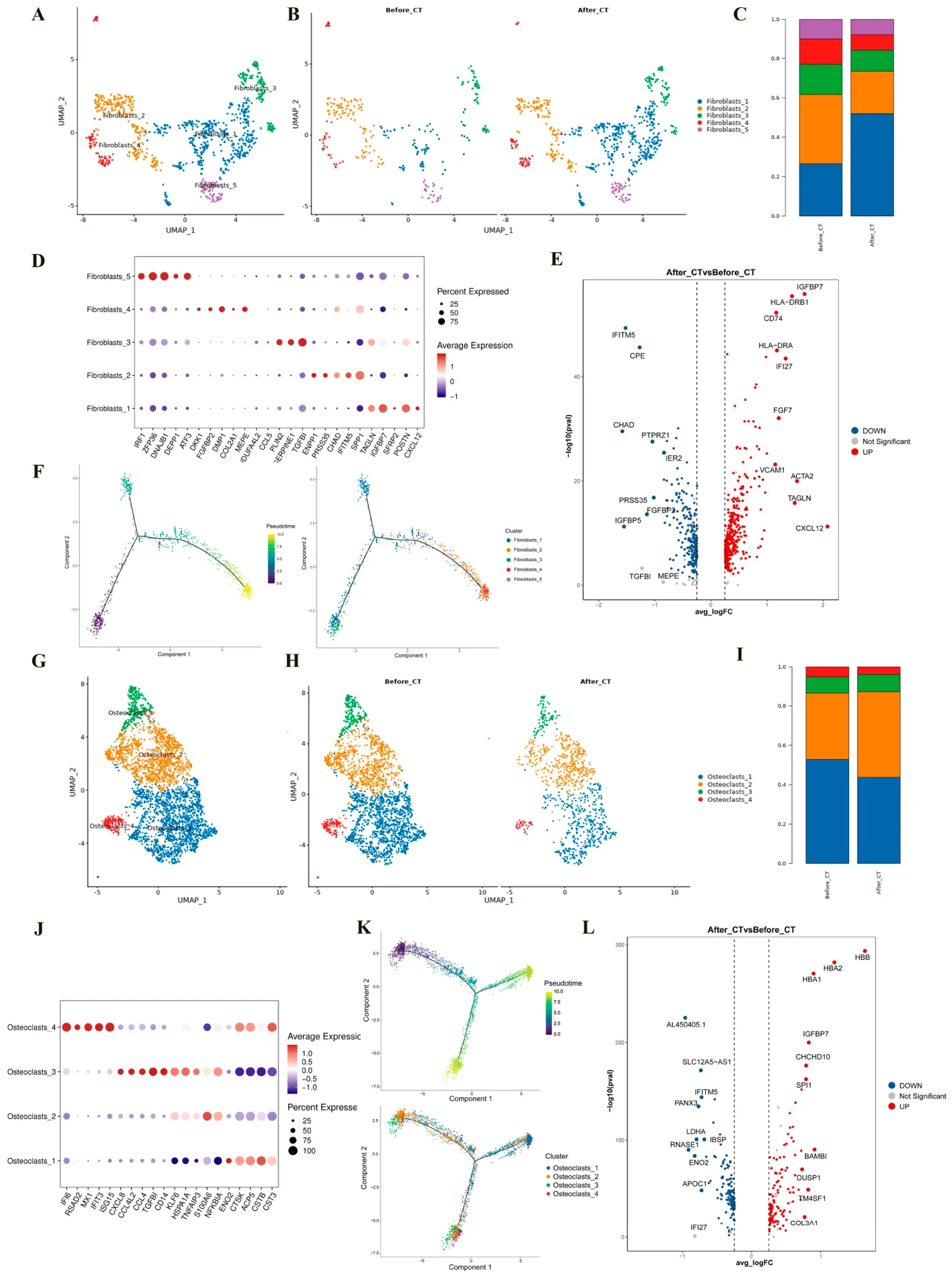
Fibroblast and osteoclast subpopulation segmentation and proposed time series analysis. (**A**) UMAP plot showing fibroblast subpopulations. (**B**) UMAP plot showing different fibroblast types before and after NACT. (**C**) Bar graph of fibroblast type ratios before and after NACT. (**D**) Bubble map of fibroblast type marker genes. (**E**) Display of differentially expressed genes before and after NACT in osteoclasts showing only the top 10 up- and down-regulated genes. (**F**) Display the proposed chronological diagram of fibroblasts, the sequence of time indicates the sequence of pseudo-times of differentiation. (**G**) UMAP plot showing osteoclast subpopulations. (**H**) UMAP plot showing different osteoclast types before and after NACT. (**I**) Bar graph of osteoclast type ratios before and after NACT. (**J**) Bubble map of osteoclast type marker genes. (**K**) Display the proposed chronological diagram of osteoclasts, the sequence of time indicates the sequence of pseudo-times of differentiation, and distribution of different osteoclast types with pseudo-time before and after NACT. (**L**) Display of differential genes before and after NACT in osteoclasts, showing only the top 10 up- and down-regulated genes.

Fibroblasts were confirmed via *DCN*, *LUM*, and *COL1A1* expression, and 923 cells were selected for re-clustering analysis. These 923 fibroblasts were classified into five subtypes based on the expression of marker genes in each cell subpopulation: fibroblasts_1 (*CXCL12*, *POSTN*, and *IGFBP7*), fibroblasts_2 (*SPP1* and *ENPP1*), fibroblasts_3 (*TGFBI*, *SERPINE1*, and *PLIN2*), fibroblasts_4 (*MEPE* and *DMP1*), and fibroblasts_5 (*ZFP36* and *DNAJB1*) (Figure 7A). The marker gene details for each cell type were shown in Figure 7D. The UMAP plot showed the fibroblast subpopulations in the two groups, colored to show cluster information. As shown in the UMAP, fibroblasts became more abundant after NACT, especially fibroblasts_1 (Figure 7B,C). The volcano plot of DEGs between the groups was created by plotting the log-fold change in average expression against the difference in expression percentages across the two clusters (Figure 7E). To further explore the phenotypic change mechanism in fibroblasts, pseudo-time analysis was performed to infer their developmental trajectory using Monocle. Fibroblasts_1 was the starting point of this cell trajectory, finally amounting to fibroblasts_2 and fibroblasts_4 (Figure 7F). The entire trajectory was divided into two segments based on branch point 1 and named states 1 and 2 according to their developmental order (Figure S2B). Expression levels of fibrosis marker genes progressively increased in these cells (Figure S2C).

To obtain a more comprehensive understanding of the types of osteoclasts in OS, four different types were identified based on the UMAP algorithm (Figure 7G). Osteoclasts are multinucleated giant cells derived from precursor cells of hematopoietic macrophage/monocyte lines [36,37]. The cell numbers and proportions of these four osteoclast subclusters showed notable variation between the two groups, especially osteoclasts_1 and osteoclasts_2 (Figure 7H,I), suggesting that their function is a key TME component after NACT. Osteoclasts_1 showed distinct expression of mature osteoclast markers, including *CTSK* and *ACP5* (Figure 7J). We also performed a trajectory analysis of the osteoclasts based on the Monocle 2 algorithm to infer their maturation course in the OS lesions (Figure 7K). The trajectory algorithms showed that the gene expression signature patterns were consistent with the subcluster distribution identified via the UMAP plots. The differentiation state and trajectory of each osteoclast subgroup changed after NACT. Next, we analyzed DEGs comparing the transcriptomes in different groups. Hemoglobin subunits (*HBA1*, *HBA2*, and *HBB*) were upregulated after NACT (Figure 7L).

### 3.8. Construction of the Single-Cell Network Before and After NACT

**Figure 8:**
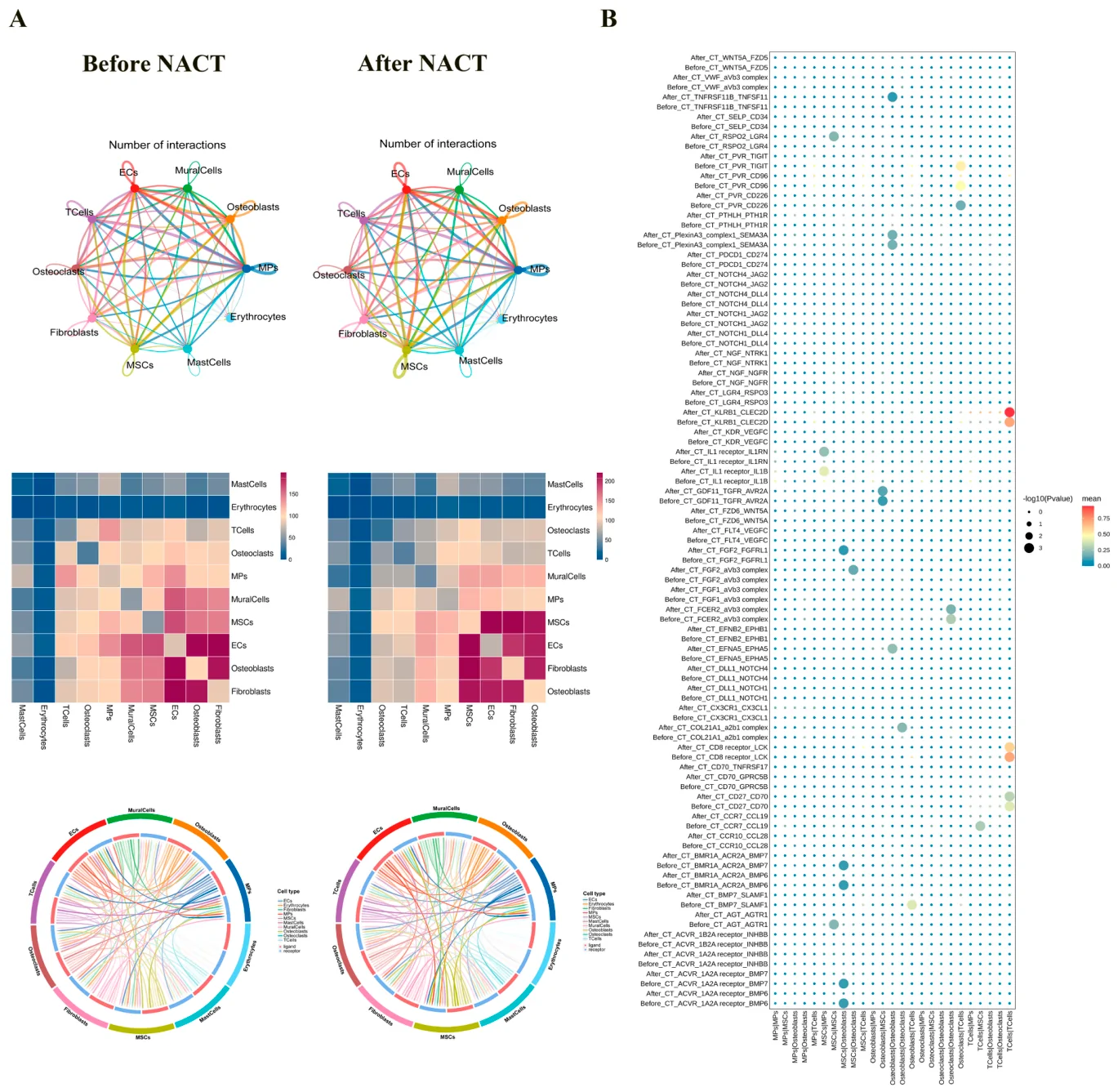
Cell communication analysis. (**A**) Net plot, heatmap and chord plot summarizing interconnections between different cell types before and after NACT. Line thickness represents the number of predicted ligand–receptor interactions; heatmap color intensity indicates interaction strength. (**B**) The molecular interaction pairs of different cell type interaction pairs.

To describe the molecular links underlying intercellular relationships, CellPhoneDB analysis was used to identify the molecular interactions between ligand–receptor pairs and major cell types to construct cellular communication networks. First, CellPhoneDB was used to analyze the cellular communication among all cell types. The results revealed the number of ligand receptors in all cell types (Figure 8A). ECs and stromal cells, particularly fibroblasts and osteoblasts, interacted with other cell types. Among the immune cells, MPs were most extensively connected to other cells. After NACT, communication between all cells increased, especially between MSCs. Immune cells, especially T cells and monocytes, played a major role in the TME of the NACT group. In general, the number of predicted interactions between tumor and immune cells decreased after treatment, whereas the interactions between MSCs and tumor cells increased. These findings suggest that a new immune microenvironmental balance emerges after chemotherapy.

To investigate the putative cell–cell interaction heterogeneity in OS subtypes, we employed a set of ligand–receptor pairs to gain insight into the regulatory relationships among all cell types identified in our study. A total of 529 and 502 pairs were predicted to mediate the interactions in all tumor tissue cells before and after NACT, respectively (Figure 8B).

Next, analysis of the secreted ligands for the detected cognate receptors demonstrated extensive communication between the different cell types in the TME (Figure 8B). Interactions between tumor cells and T cell subsets via BMP7_SLAMF1 decreased after NACT, suggesting a reduction in recruitment and co-stimulation interactions following NACT. The IL1 receptor_IL1B interaction scores between tumor cells and MPs decreased after NACT. In contrast, interactions between tumor cell and MSCs subsets via GDF11_TGFR_AVR2A increased after NACT.

## 4. Discussion

OS remains a formidable malignancy characterized by aggressive progression and dismal prognosis, particularly in advanced stages. Current therapeutic strategies such as NACT often yield limited efficacy due to the tumor’s heterogeneous cellular composition and intricate interactions within the TME. To address this therapeutic impasse, it is imperative to comprehensively dissect OS phenotypes and their microenvironmental dynamics. In this study, we employed high-resolution scRNA-seq to delineate the cellular heterogeneity of the OS microenvironment and its differential response to chemotherapy. Unlike in previous studies, we utilized samples collected both before and after NACT, profiling a total of 77,616 cells from 12 OS patients. Our results unveiled remarkable complexity, identifying distinct cellular subpopulations—including osteoblasts, macrophages, and endothelial cells—each exhibiting unique transcriptional profiles and discrete responses to NACT. Notably, significant post-treatment alterations in cell abundance and gene expression patterns were observed, underscoring the pivotal role of the TME in modulating therapeutic efficacy. These findings provide a comprehensive landscape of OS cellular dynamics and, importantly, highlight the intricate intercellular communication within the TME as a key determinant of treatment response.

Specifically, among the twelve patients who received NACT (DOX + DDP + MTX), six exhibited low cell viability and high impurity density in surgical specimens, which were attributed to post-treatment calcification, indicating chemosensitivity. In contrast, malignant osteoblasts were detectable in the remaining six patients, especially in sample B5; therefore, we compared these samples with the other treated samples. Pathway enrichment revealed that upregulated genes in the resistant samples were significantly enriched in the PI3K-AKT pathway, a well-known oncogenic cascade implicated in various tumors, including OS [38,39,40]. This finding aligns with previous reports that PI3K/Akt activation promotes tumor cell proliferation, apoptosis resistance, and angiogenesis [41,42]. Moreover, studies have shown that PI3K/Akt inhibitors can reverse multidrug resistance [43,44]. In addition to PI3K-AKT signaling, another key mechanism driving treatment resistance is EMT. EMT confers tumor cells with MSC-like characteristics and adaptive plasticity, enabling rapid adaptation to stress and fostering a treatment-resistant phenotype [45]. Its activation is orchestrated by complex signaling networks, including the Wnt, Hedgehog, TGF-β, NF-κB, and Notch pathways, which often cooperate to sustain EMT [46,47]. Among these, the Notch signaling pathway has been implicated in both EMT and drug resistance in various cancers, such as colorectal and ovarian cancer [48,49]. Our scRNA-seq analysis now provides the first evidence that EMT-related pathways are enriched and associated genes are highly expressed in specific osteoblast subsets from chemoresistant OS patients, suggesting that an EMT program may be activated in OS cells. Together with the PI3K-AKT activation, these observations reveal the multifaceted nature of chemoresistance in OS and highlight potential therapeutic targets beyond intrinsic tumor alterations. However, previous studies on OS have mainly focused on the molecular biological changes in tumor cells themselves, while largely overlooking the contribution of the non-tumor cell microenvironment.

Tumor-infiltrating myeloid cells, particularly TAMs, are crucial and abundant components of the TME. Monocytes in the blood are recruited to the TME and become TAMs based on internal environment conditions, such as the presence of chemokines, cytokines, and other factors secreted by tumor cells, MSCs, and immune cells, as well as local anoxia, inflammation, or high lactic acid levels [50]. Numerous studies have established a close relationship between high TAM density and poor prognosis across various malignancies, including OS [51,52]. Despite their recognized importance, the precise mechanisms by which TAMs are recruited and reprogrammed within the OS TME—especially in the context of NACT—remain poorly understood. Bruchard et al. [53] found that cathepsin derived from myeloid cells promotes IL-1β secretion, which in turn stimulates IL-17 secretion from CD4+ T cells, thereby weakening the anticancer effects of chemotherapy. This highlights the complex, multi-step nature of TAM-driven resistance, which cannot be fully explained by a single axis. Interestingly, our data also revealed IL-17 pathway enrichment in multiple macrophage subsets from post-treatment subgroups, suggesting that IL-17 signaling may also play a role in the NACT-induced immune modulation of OS, though its specific contribution requires further investigation. Recently, the role of ECs in shaping the immune microenvironment via chemokine signaling has garnered increasing attention. However, the specific molecular mediators of EC–macrophage crosstalk in the NACT-treated OS TME have yet to be fully elucidated. Given that chemokines are among the principal drivers of monocyte/macrophage recruitment, we asked whether EC-derived chemokine signaling represents the missing molecular link governing macrophage recruitment within the post-NACT TME.

To address this question, we systematically examined the chemokine signaling landscape of the OS TME before and after NACT and demonstrate that NACT profoundly reshapes this network. Notably, ECs exhibited significant expression of *CXCL2*, and its expression levels were markedly altered after NACT, suggesting that NACT may reshape the chemokine network to influence the immune microenvironment and potentially induce stromal-mediated resistance. Among its receptors, *CXCR2* was identified as the predominant receptor on macrophages, and its expression was significantly upregulated in macrophages following NACT. Heatmap analysis further revealed strong correlations between macrophage *CXCR2* and multiple EC-derived ligand genes before and after treatment, supporting the central role of the CXCL2–CXCR2 axis in intercellular communication. Functional validation assays demonstrated that recombinant CXCL2 directly enhanced macrophage migration, and ECs overexpressing *CXCL2* effectively promoted macrophage chemotaxis, confirming the functional activity of endothelial-derived CXCL2. Western blot analysis confirmed corresponding changes at the CXCR2 protein level. Previous studies have shown that the CXCL2–CXCR2 signaling axis plays a critical role in the recruitment and protumor polarization of TAMs [54], and chemotherapy-induced upregulation of *CXCL2* may exacerbate this process, thereby affecting treatment efficacy [55]. Notably, in gastric cancer, melanoma, lung cancer and others, blocking CXCR2 enhanced chemosensitivity and suppressed tumor metastasis [56,57]. Collectively, our findings suggest that NACT upregulates CXCL2 in ECs, which in turn activates CXCR2 signaling in macrophages, promoting recruitment of pro-inflammatory or immunosuppressive macrophages into the tumor region. This dynamic regulatory mechanism may provide new insights into chemoresistance and immune evasion, and targeting the CXCL2–CXCR2 axis could represent a potential strategy to improve NACT efficacy. In addition, after NACT, immune signaling pathways, such as interferon-alpha and interferon-gamma responses, were significantly enriched in vascular ECs, further highlighting the multifaceted role of ECs in shaping the TME and therapeutic response. These observations suggest that NACT-induced immune pathway activation in ECs may contribute to dynamic TME changes, though the downstream functional consequences—such as whether they synergize with or counteract the CXCL2-driven resistance—require further investigation. However, further in vivo studies are needed to validate the causal role of this axis and to elucidate downstream signaling pathways and corresponding immune phenotypic changes.

Collectively, our results identify the EC-derived CXCL2–CXCR2 axis as a novel mediator of intercellular communication in the OS TME that is dynamically regulated by NACT. This axis may represent a critical mechanism contributing to NACT-induced stromal-mediated resistance and immune evasion. Targeting this pathway, specifically by blocking *CXCR2*, could offer a promising strategy to enhance the efficacy of NACT in OS patients. Nevertheless, the present study is primarily correlative and requires further functional validation. Future in vivo studies are needed to causally establish the role of this axis in chemoresistance and to investigate the downstream signaling cascades and corresponding changes in the immune landscape. In addition, our scRNA-seq analysis revealed that fibroblast subclusters (notably fibroblasts_1) and osteoclast subclusters (especially osteoclasts_1 and osteoclasts_2) also underwent significant compositional and trajectory changes after NACT. These findings underscore the complexity of the NACT-remodeled TME, and future studies should explore whether these stromal populations interact with osteoblasts, ECs or macrophages to modulate chemoresistance.

We acknowledge several important limitations in this study. The absence of matched patient samples before and after NACT limits our ability to directly track clonal dynamics and TME evolution within individual tumors. Future studies utilizing paired biopsies will be crucial for establishing a more precise, intra-patient timeline of these immune-stromal changes. Additionally, the lack of longitudinal clinical follow-up data, such as progression-free or overall survival, restricts the assessment of the prognostic and predictive significance of the EC-derived CXCL2–CXCR2 axis. Systematic correlation of the identified signaling alterations with clinical outcomes in larger, prospectively followed patient cohorts is a necessary next step. Despite these constraints, our study provides novel and foundational insights into the NACT-remodeled TME in OS, highlighting the CXCL2–CXCR2 axis as a promising therapeutic target and paving the way for more targeted functional and translational studies.

## 5. Conclusions

In this study, we leveraged single-cell RNA sequencing to systematically characterize the NACT-remodeled OS microenvironment. We identify the endothelial-derived CXCL2–CXCR2 axis as a critical mediator of macrophage recruitment, contributing to stromal-driven chemoresistance and immune evasion. Furthermore, the enrichment of PI3K-AKT and EMT pathways in resistant osteoblasts underscores the multifaceted nature of treatment failure. These findings highlight intercellular communication as a key determinant of therapeutic response and position the CXCL2–CXCR2 axis as a promising target to enhance NACT efficacy. Future functional studies with paired patient samples and longitudinal follow-up are warranted to translate these insights into clinical benefit.

## Data Availability

The datasets used and/or analyzed during the current study are available from the corresponding author on reasonable request.

## Supplementary Materials

The following supporting information can be downloaded at: https://www.mdpi.com/article/10.3390/biomedicines14091966/s1, Figure S1: Overview of the single-cell transcriptomic landscape and characterization of identified cell types; Figure S2: Analysis of cell proportions and pseudotime dynamics of gene expression; Table S1: Patient demographics, treatment status, and scRNA-seq quality of 18 osteosarcoma samples from naive and NACT cohorts.

## Abbreviations

OS	osteosarcoma
NACT	neoadjuvant chemotherapy
scRNA-seq	single-cell RNA sequencing
TME	tumor microenvironment
MTX	methotrexate
DOX	doxorubicin
CXCL2	C-X-C motif chemokine ligand 2
CXCR2	C-X-C motif chemokine receptor 2
HUVECs	human umbilical vein endothelial cells
THP-1	human acute monocytic leukemia cell line
DDP	cisplatin
RCLB	red blood cell lysis buffer
UMI	unique molecular identifier
DEG	differentially expressed gene
GO	Gene Ontology
KEGG	Kyoto Encyclopedia of Genes and Genomes
GSVA	gene set variation analysis
ECs	endothelial cells
MPs	mononuclear phagocytes
MSCs	mesenchymal stem cells
UMAP	uniform manifold approximation and projection
CapEC	capillary endothelial cell
VEC	venule endothelial cell
SCENIC	single-cell regulatory network inference and clustering
TF	transcription factor
CNV	copy number variation
EMT	epithelial–mesenchymal transition
TAM	tumor-associated macrophage
PIC	piceatannol
