# Supplementary material for: Chemokine-Driven Intercellular Crosstalk in the Osteosarcoma Microenvironment After Neoadjuvant Chemotherapy: A Single-Cell RNA Sequencing Study
Source: Biomedicines. 2026 Aug 31;14(9):1966. doi: 10.3390/biomedicines14091966 (PMC13604099; doi:10.3390/biomedicines14091966)
Supplement: Supplementary file 1 [file biomedicines-14-01966-s001.zip › Supplementary Table S1.pdf]

Supplementary Table S1. Patient demographics, treatment status, and scRNA-seq quality of 18 osteosarcoma samples from naive and neoadjuvant chemotherapy (NACT) cohorts.

Sample ID	Patient ID	Age	Sex	Tumor Site	Sample Type	Treatment Status	Chemotherapy Response	Cell activity	scRNA-seq Quality
A1	625866	12	M	Left tibia	Biopsy(puncture)	Naive	Sensitive	94.99%	Success
A2	627972	14	F	Right femur	Biopsy(puncture)	Naive	Sensitive	86.42%	Success
A3	629270	12	M	Left femur	Biopsy(puncture)	Naive	Sensitive	88.76%	Success
A5	630083	46	F	Right femur	Resection(amputation)	Naive	Sensitive	82.53%	Success
A6	631911	22	M	Right tibia	Biopsy(puncture)	Naive	Sensitive	77.85%	Success
A7	631627	17	M	Right femur	Biopsy(puncture)	Naive	Sensitive	86.41%	Success
B2	607556	18	M	Left tibia	Resection	NACT	Resistant	82.81%	Success
B1	610382	29	F	Left femur	Resection	NACT	Resistant	82.61%	Success
B3	618170	20	F	Left femur	Resection	NACT	Resistant	67.07%	Success
B4	617142	9	M	Right femur	Resection	NACT	Resistant	91.29%	Success
B5	623425	32	F	Right femur	Resection	NACT	Resistant	91.84%	Success
B6	618527	16	F	Left tibia	Resection	NACT	Resistant	89.00%	Success
N/A	611996	47	M	Left femur	Resection	NACT	Sensitive	25.60%	Failure
N/A	618123	28	F	Sacrum	Resection	NACT	Sensitive	50.00%	Failure
N/A	616274	18	M	Left tibia	Resection	NACT	Sensitive	48.63%	Failure
N/A	621665	17	F	Right femur	Resection	NACT	Sensitive	39.89%	Failure

N/A	620998	19	M	Left humerus	Resection	NACT	Sensitive	57.77%	Failure
N/A	610211	10	F	Right femur	Resection	NACT	Sensitive	32.92%	Failure

Note. Cell viability < 60% was considered ineligible for scRNA-seq.
